# Portal Hypertension

**DOI:** 10.1155/2018/1289156

**Published:** 2018-11-04

**Authors:** Mingyu Sun, Xingshun Qi, Fernando G. Romeiro, Andrea Mancuso

**Affiliations:** ^1^Institute of Liver Diseases, Shuguang Hospital Affiliated to Shanghai University of Traditional Chinese Medicine, Shanghai, China; ^2^Department of Gastroenterology, General Hospital of Shenyang Military Area, Shenyang, China; ^3^Faculdade de Medicina de Botucatu UNESP, Campus de Botucatu, s/n, Botucatu, SP, Brazil; ^4^Medicina Interna 1, ARNAS-Civico, Di Cristina-Benfratelli, Palermo, Italy

A major cause of morbidity and mortality in cirrhosis is the development of variceal bleeding, a direct consequence of portal hypertension. Portal hypertension occurs when hepatic venous pressure gradient (HVPG) is above 5 mmHg, but the main complications are clinically expressed when it exceeds 10 mmHg. Ascites, gastroesophageal variceal bleeding, hepatorenal syndrome, and hepatic encephalopathy are life-threatening conditions related to portal hypertension. In some countries, the economic burden caused by liver diseases and portal hypertension complications is higher than that of chronic kidney disease and type 2 diabetes combined. Most cases of portal hypertension in the Western world are attributed to liver cirrhosis (90% of patients). Structural changes such as depots of fibrous tissue, microthrombi, collapse of the liver parenchyma, and vascular alterations account for approximately 70% of the hepatic vascular resistance observed in portal hypertension due to cirrhosis.

This special issue aims to bring innovative articles on this matter, given to the readers an integrative point of view about the hot topics on diagnostic and treatment of portal hypertension complications. The articles received by the journal were carefully selected, thus offering a high-quality collection.

A study by H. Li et al. explored the association between the pathological damage of mitochondrial ultrastructure according to the Flameng classification system and risk of hepatic encephalopathy in patients treated with transjugular intrahepatic portosystemic shunt. The investigators provided invaluable data from the transjugular liver biopsy procedures. Based on the histological and clinical data, the investigators reported interesting findings that more severe damage to mitochondrial ultrastructure was significantly associated with a higher risk of hepatic encephalopathy after transjugular intrahepatic portosystemic shunt. Additionally, they found that the severity of damage to mitochondrial ultrastructure positively correlated with the venous ammonia level. These findings would be useful to deeply understand the mechanisms of developing hepatic encephalopathy after transjugular intrahepatic portosystemic shunt and to establish the preventive strategy in future.

The noninvasive serological scoring system has become a research hotspot in predicting hepatic fibrosis. F. Zhang et al. contributed their research paper “Predictive Value of a Noninvasive Serological Hepatic Fibrosis Scoring System in Cirrhosis Combined with Oesophageal Varices”. They retrospectively analysed cirrhosis patients with or without oesophageal varices, aiming to evaluate the predictive value of the four following scoring systems in cirrhosis combined with oesophageal varices: aspartate and platelet ratio index (APRI), aspartate aminotransferase-alanine aminotransferase ratio (AAR), FIB-4, and S index. A total of 153 patients with cirrhosis were categorized into groups with or without oesophageal varices. AAR harbored a poor predictive value for oesophageal varices, APRI can be used as a reference index for the prediction of severe oesophageal varices, and the S index harbored potential value in predicting the degree of progression of cirrhosis.

X. Qi et al. review paper “Transient Elastography for Significant Liver Fibrosis and Cirrhosis in Chronic Hepatitis B” performed the meta-analysis describing diagnostic accuracy of transient elastography (TE) for predicting CHB-related fibrosis. Hierarchical summary receiver operating curves model and the bivariate random efforts model were applied to generate summary receiver operating characteristic curves and pooled estimates of sensitivity and specificity. TE is of great value in the detection of patients with CHB-related cirrhosis but has a suboptimal accuracy in the detection of significant fibrosis.

D. Kara et al. presented a paper on portal hypertensive polyposis in advanced liver cirrhosis aiming to identify relevant endoscopic findings in patients with advanced cirrhosis and consecutive portal hypertension. This retrospective study on liver transplant candidates undergoing 1,045 upper endoscopies found that portal hypertensive gastric and duodenal polyps were frequently observed and were significantly associated with thrombocytopenia, Child-Pugh score, MELD, and previous rubber band ligation. These polyps often recurred after polypectomy; however, no malignant transformation occurred during observation. The most common endoscopic finding was esophageal varices, observed in more than 90% of patients. The authors concluded that portal hypertensive polyposis is common in patients with advanced cirrhosis and these polyps are generally benign.

X. Luo et al. prepared a paper on the “Short-Term Outcome of Patients with Cirrhosis and Concurrent Portal Cavernoma Presenting with Acute Variceal Bleeding” in which they compared short-term outcomes after acute variceal bleeding (AVB) in cirrhotics with and without portal cavernoma, finding that the 5-day treatment failure rate was higher in the cavernoma group than in the control group (32.1% versus 12.5%). The 6-week mortality rate did not differ significantly between the cavernoma and control group (25% versus 12.5%). Multivariable Cox proportional hazard regression analyses revealed that 5-day treatment failure independently predicted 6-week mortality.

In another interesting paper written by Tseng et al., the authors present their own experience facing severe adverse events after endoscopic cyanoacrylate injection. The authors also made a review on this issue, looking for findings that could be associated with an increased risk in this procedure, thus providing a further insight into the study. Since cyanoacrylate injection is the first line therapy for variceal bleeding from gastric varices, the readers have the chance of knowing the authors' experience. For instance, they have used some pre- and posttreatment medications and their protocol applies the sandwich technique injection of lauromacrogol and cyanoacrylate. Furthermore, the article has detailed information on the patient's conditions before the procedure, including some findings that are lacking in prior studies. It is a remarkable advantage of this paper, presenting some hints on how to predict severe adverse effects after cyanoacrylate injection.

